# Replication of Epstein-Barr Virus Primary Infection in Human Tonsil Tissue Explants

**DOI:** 10.1371/journal.pone.0025490

**Published:** 2011-10-05

**Authors:** Kensei Gotoh, Yoshinori Ito, Seiji Maruo, Kenzo Takada, Terukazu Mizuno, Masaaki Teranishi, Seiichi Nakata, Tsutomu Nakashima, Seiko Iwata, Fumi Goshima, Shigeo Nakamura, Hiroshi Kimura

**Affiliations:** 1 Department of Pediatrics, Nagoya University Graduate School of Medicine, Nagoya, Japan; 2 Department of Tumor Virology, Institute for Genetic Medicine, Hokkaido University, Sapporo, Japan; 3 Department of Otorhinolaryngology, Nagoya University Graduate School of Medicine, Nagoya, Japan; 4 Department of Virology, Nagoya University Graduate School of Medicine, Nagoya, Japan; 5 Department of Pathology and Laboratory Medicine, Nagoya University Hospital, Nagoya, Japan; University of Nebraska – Lincoln, United States of America

## Abstract

Epstein-Barr virus (EBV) may cause a variety of virus-associated diseases, but no antiviral agents have yet been developed against this virus. Animal models are thus indispensable for the pathological analysis of EBV-related infections and the elucidation of therapeutic methods. To establish a model system for the study of EBV infection, we tested the ability of B95–8 virus and recombinant EBV expressing enhanced green fluorescent protein (EGFP) to replicate in human lymphoid tissue. Human tonsil tissues that had been surgically removed during routine tonsillectomy were sectioned into small blocks and placed on top of collagen sponge gels in culture medium at the air-interface, then a cell-free viral suspension was directly applied to the top of each tissue block. Increasing levels of EBV DNA in culture medium were observed after 12–15 days through 24 days post-infection in tissue models infected with B95–8 and EGFP-EBV. Expression levels of eight EBV-associated genes in cells collected from culture medium were increased during culture. EBV-encoded small RNA-positive cells were detected in the interfollicular areas in paraffin-embedded sections. Flow cytometric analyses revealed that most EGFP^+^ cells were CD3^−^ CD56^−^ CD19^+^ HLA-DR^+^, and represented both naïve (immunoglobulin D^+^) and memory (CD27^+^) B cells. Moreover, EBV replication in this model was suppressed by acyclovir treatment in a dose-dependent manner. These data suggest that this model has potential for use in the pathological analysis of local tissues at the time of primary infection, as well as for screening novel antiviral agents.

## Introduction

Epstein-Barr Virus (EBV) is a universal human γ-herpesvirus, generally transmitted via saliva, with the oropharynx as the site of infection [1], [2]. Primary EBV infection occurs most frequently in infancy and childhood, and in many cases causes either no or only nonspecific symptoms. In cases of primary infection among adolescents and young adults, infectious mononucleosis (IM) often develops, and the course may sometimes be severe or fatal. After infection, EBV remains in most adults as an asymptomatic latent infection, but may cause neoplastic disorders such as Burkitt's lymphoma or post-transplant lymphoproliferative disorder (PTLD).

Although EBV may cause a variety of disorders, no vaccine or antiviral agent has yet been developed against this virus [1], [2]. In general, animal models are indispensable for the pathological analysis of viral infections and the elucidation of methods of treatment and prevention, but EBV only infects humans in nature and limited animal species under experimental conditions. Various infection models have been used to investigate EBV-associated diseases [3], [4], [5], [6], [7]. Mouse models that partially reconstitute human immune system components after engagement of hematopoietic progenitor cells are of particular interest, because they reproduce human immunity and diseases caused by EBV. Several mouse models of immunodeficiency have been applied, including Rag2^−/−^ γc^−/−^ mice [8], [9], NOD/SCID mice [10], NOD/SCID/γc^−/−^ mice [11], [12], [13], BLT mice (NOD/SCID mice with implantation of human fetal liver and thymus pieces under the renal capsule) [14], and NOD/Shi-SCID/IL-2Rγ^null^ (NOG) mice [15]. Of these, the NOG mice model [15] has been used to show that B-cell lymphoproliferative disorder arises during EBV infection with a high viral load, whereas asymptomatic persistent infection arises from infection with a low viral load. In addition, EBV-specific T-cell responses and EBV-specific antibodies were detected in blood, revealing this mouse model as a useful tool for investigating the pathogenesis, prevention, and treatment of EBV infection. Culture models using human lymphatic tissues, however, appear advantageous for the study of localized pathology in EBV infection. We therefore focused on a model of infection using human tonsillar lymphoid tissues.

Numerous reports have described the use of viral infection models using human tonsillar lymphoid tissues for the study of human immunodeficiency virus (HIV) [16], while others have described the use of such models for investigating other members of the herpesvirus family, such as human herpesvirus (HHV)-6, HHV-7, and herpes simplex virus (HSV)-2 [17], [18], [19]. The palatine tonsils comprise typical lymphoid tissue and are also the natural portal of entry for EBV, thus showing great potential for reproducing the pathology of primary infection with EBV. The present study used human tonsillar lymphoid tissues to establish an EBV infection model and investigated infected cells during the initial stage of infection. We also tested the utility of this model as a screening system for antiviral agents.

## Materials and Methods

### Ethics statement

Human subject protocols were approved by the institutional review board of Nagoya University School of Medicine (2006-450). Written informed consent was provided by study participants and/or their legal guardians prior to enrolment.

### Virus stocks

Cell-free virus solution was obtained from culture supernatant of B95–8 cells (an EBV-infected marmoset cell line) (ATCC) after centrifuging for 5 min at 3,000×*g* and filtration through a 0.45-µm membrane filter. In some experiments, we also used supernatants containing recombinant EBV expressing enhanced green fluorescent protein (EGFP), in which a gene cassette consisting of the EGFP gene driven by the simian virus 40 promoter and a neomycin resistance gene driven by the simian virus 40 promoter were inserted into the viral BXLF1 gene [20]. Titers of virus in the 50% transforming dose (TD_50_) were determined using the Reed-Muench method, as described elsewhere [15], [21]. Calculated titers were 1×10^2^ TD_50_/ml for B95 and 1×10^3^ TD_50_/ml for EGFP-EBV.

### Tissue culture and viral infection

Human tonsils surgically removed during routine tonsillectomy and not required for clinical purposes were received within several hours of excision, then dissected, cultured, and infected, as described elsewhere [16], [22], [23]. Since EBV-specific immune responses may impact cellular infection, we only used tonsils from EBV-seronegative individuals. Serological tests for anti-EBV antibodies were examined 2–4 weeks preoperatively and negative results for viral capsid antigen immunoglobulin (Ig) G and EBV nuclear antigen were confirmed. In brief, the tonsils were washed thoroughly with medium containing antibiotics, sectioned into cubes with an average weight of 5 mg, and placed on top of collagen sponge gels in culture medium at the air-interface. For EBV infection, 10 µL of clarified viral suspension was directly applied on top of each tissue block. The culture medium used to bathe 54 tissue blocks in six wells was collected every 3 days after viral inoculation. Cell-free supernatants and cells were obtained after centrifugation of collected medium and used for the following assays. Cells collected from culture medium were considered to have come from infected tissues. Most of the epithelial tonsillar layers were lost during preparation of tissue blocks, so replication of EBV in epithelial cells could not be examined.

### Quantification of EBV DNA

Viral DNA was extracted from either 200 µL of cell-free culture supernatants or from 5×10^5^ cells collected from culture medium using QIAamp DNA blood kits (Qiagen). Real-time quantitative PCR assays were performed as previously described [24], [25], [26].

### RNA purification and real-time RT-PCR

RNA was extracted from 5×10^5^ cells from culture medium with a QIAmp RNeasy Mini Kit (Qiagen). Viral mRNA expression was quantified by one-step multiplex real-time reverse transcription (RT)-PCR using the Mx3000P real-time PCR system (Stratagene), as described previously [27], [28], to examine expression levels of two lytic genes (BZLF1 and gp350/220) and six latent genes (EBV-encoded nuclear antigen (EBNA)1, EBNA2, latent membrane protein (LMP)1, LMP2, EBV-encoded small RNA (EBER)1, and *Bam*HI-A rightward transcripts (BARTs)). The stably expressed housekeeping gene β_2_-microglobulin (*β2M*) was used as an endogenous control and reference gene for relative quantification. Each experiment was conducted in triplicate and results are shown as the mean of three samples with standard errors.

### Immunohistology and *in situ* hybridization

Tissue blocks were fixed with 10% buffered formalin, embedded in paraffin, and sectioned at 5 µm and stained with hematoxylin and eosin (HE). The monoclonal antibodies used were anti-CD3, anti-CD20, anti-follicular dendritic cell (FDC), EBNA2, LMP1 and BZFL1 (Dako), all of which were used after antigen retrieval following heating in a microwave oven [27], [29]. *In situ* hybridization was performed using the EBER1 probe (Dako) as previously described [24], [27]. Hybridization was detected using mouse monoclonal anti-fluorescein isothiocyanate (Dako) and a Vectastain ABC kit (Vector). For both immunostaining and *in situ* hybridization, diaminobenzidine was used for visualization.

### Flow cytometry

At day 15–24 post-infection with EGFP-EBV, single-cell suspensions were dissected from tissue blocks by mechanical dissociation. Tissue blocks were placed into a petri plate with complete medium and gently ground with pestles. As shown previously, this procedure releases lymphocytes from stromal elements [23]. Similarly, cells that had emigrated into the collagen sponge were mechanically squeezed out and collected by centrifugation. Cell debris and fragments were removed and mononuclear cells (MNCs) were purified by Ficoll-Hypaque centrifugation [30]. MNCs were washed three times and stained using a combination of the following monoclonal antibodies (mAbs): phycoerythrin (PE)-labeled anti-CD19 (clone HD37; Dako); anti-CD3 (clone UCHT1; eBioscience); anti-CD56 (clone IM2073; Beckman Coulter); PE-cyanin 5 (PC5)-labeled anti-CD56 (clone IM2654; Beckman Coulter); and anti-HLA-DR (clone IMMU357; Beckman Coulter). In a few experiments, collected cells were also stained with 7-amino-actinomycinD (7-AAD; BD Pharmingen) at day 24, to exclude dead cells on samples. Discrimination between infection with memory or naive B cells was determined using the following antibody combination: PE-labeled anti-CD27 (clone M-T271; Becton Dickinson); anti-IgD (clone IgD26; Miltenyi Biotec); and PC5-labeled anti-CD19 (clone HIB19; eBioscience). Isotype-matched monoclonal mouse IgG antibodies were used in each experiment as controls. Stained cells were analyzed using FACSCalibur and CellQuest version 5.2.1 software (Becton Dickinson).

### Acyclovir (ACV) treatment

To test the anti-EBV activity of ACV (GlaxoSmithKline), B95-infected tissue blocks were incubated with culture medium containing ACV at various concentrations (1.5, 5, 15 or 45 µg/ml). Culture medium was changed every 3 days after viral inoculation, and ACV was added with every medium change. A sample of medium collected from each exchange was used for quantification of EBV DNA, as previously mentioned.

### Flow cytometric in situ hybridization (FISH) assay

To quantify EBV-infected cells and to analyze the cell types of EBV-infected populations, FISH assays were used [31]. Briefly, 5×10^5^ MNCs were stained with phycoerythrin cyanine 5 (PC5)-labeled anti-CD45 (clone HI30; Biolegend) monoclonal antibodies for 1 h at 4°C. Isotype-matched monoclonal mouse IgG antibodies were used as controls. After staining with antibodies, cells were fixed with 1% acetic acid/4% paraformaldehyde, permeabilized with 0.5% Tween 20/phosphate-buffered saline, and hybridized with a fluorescein-labeled EBER-specific peptide nucleic acid probe (Y5200; Dako). Fluorescence intensity was enhanced using the AlexaFluor 488 Signal Amplification Kit (Molecular Probes), and stained cells were analyzed using FACSCalibur and CellQuest software (BD Biosciences).

### Statistical analyses

Data are presented as means ± standard error of the mean. Statistical analyses were conducted using StatView version 5.0 software (SAS Institute). The Kruskal-Wallis test was used to compare inhibition rates of EBV infection at each concentration of ACV. Values of *P*<0.05 were considered statistically significant.

## Results

### Replication of EBV B95–8 in human tonsil tissue explants

**Figure 1:**
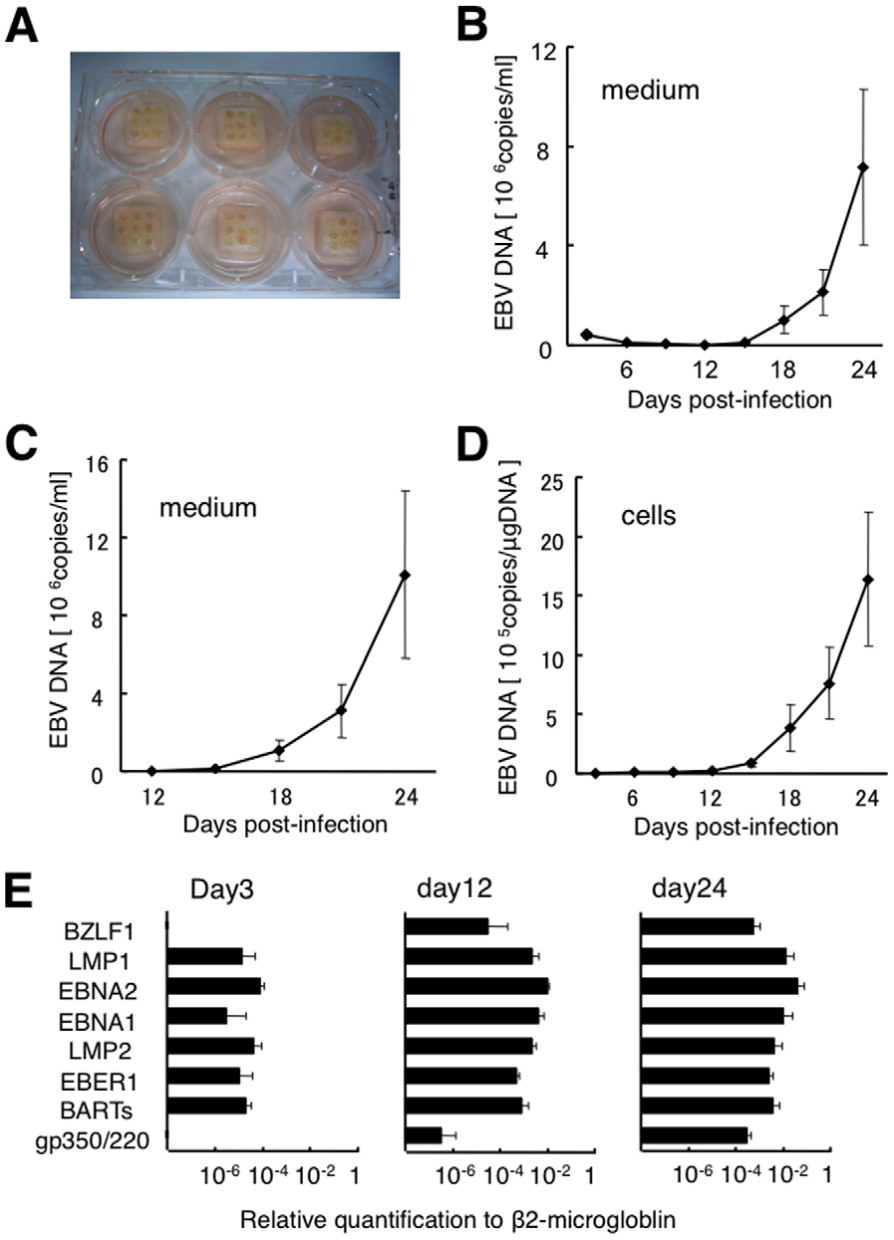
Kinetics of EBV DNA and expression patterns of EBV-related genes in human tonsil tissue explants infected with B95–8. Culture medium was changed every 3 days, and collected medium was centrifuged. Cell-free supernatants and cells collected from culture medium were used for quantification of EBV DNA by real-time PCR assay and quantification of viral mRNA by real-time RT-PCR assay. Data are presented as mean ± standard error of the mean. **A**) Tissue blocks on top of collagen sponge gels in a six-well plate. **B**) Kinetics of EBV DNA in cell-free supernatants. Average data were obtained from tissues derived from 12 donors. **C**) Plots of accumulated EBV DNA in cell-free culture medium after day 12 post-infection (*n* = 12). **D**) Kinetics of EBV DNA in cells from medium (*n* = 12). **E**) Levels of EBV-related gene expressions in cells at 3, 12, and 24 days post-infection (*n* = 4).

To establish a model system for the study of EBV infection, we tested the ability of B95–8 virus to replicate in human tonsillar tissue. The prepared tonsil tissue blocks (**Fig. 1A**) were exposed to B95–8, and cell-free supernatants and cells were collected. **Figure 1B** shows the kinetics of EBV DNA in culture supernatants of infected tissues at each time point. EBV DNA level gradually dropped and was minimal on day 12 post-infection, then steadily increased. When we applied the viral suspension on top of collagen sponge gels without tonsil tissues and changed the culture medium every 3 days, EBV DNA in culture medium gradually decreased and fell below the limit of detection by day 12 post-infection (data not shown). Kinetics before day 12 post-infection resemble those in EBV-infected tissue before day 12 post-infection, suggesting that EBV replication may become apparent after day 12 post-infection. We therefore showed the kinetics of EBV DNA in culture medium as a cumulative curve by totaling values from day 12 post-infection and after (**Fig. 1C**). Levels of viral DNA peaked between 1.3×10^5^ and 4.3×10^7^ (median, 4.1×10^6^) genome-equivalents/ml of culture supernatant on day 24 post-infection (*n* = 12). Increasing levels of EBV over time were also observed in cells from culture medium with peak levels between 1.2×10^4^ and 6.7×10^6^ (median, 8.0×10^5^) genome-equivalents/µg of DNA at day 24 post-infection (*n* = 12) (**Fig. 1D**).

Next, we analyzed expressions of eight EBV-associated genes in cells from culture medium using a real-time RT-PCR assay (**Fig. 1E**). Expression levels of all genes examined increased after day 12 post-infection (*n* = 4). The immediate early gene BZLF1 was not detected on day 3 post-infection, but was detected after day 12 post-infection. In some series of experiments, we used RNA extracted from cells from dissected tissues and measured EBV gene expression. Expression patterns of transcripts resembled those of cells collected from tissue blocks (data not shown).

### Replication of recombinant EGFP-EBV in human tonsil tissue explants

**Figure 2:**
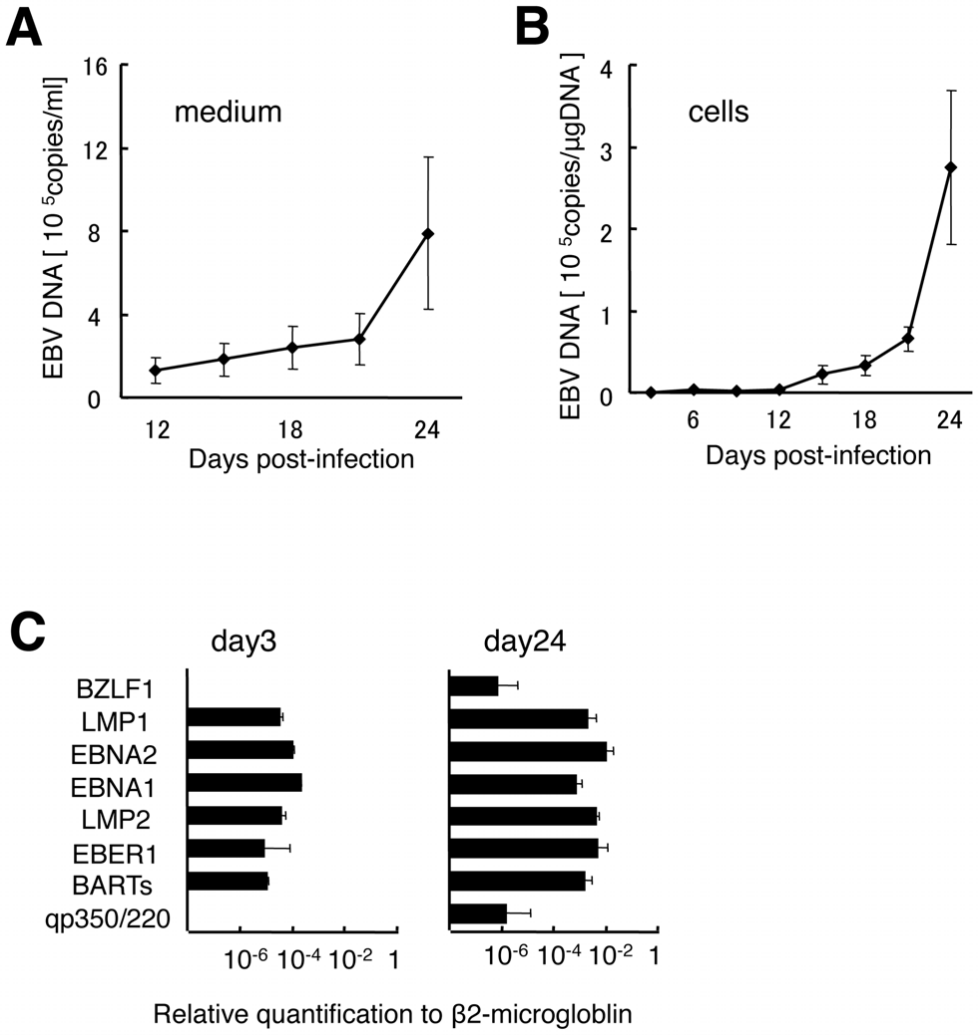
Kinetics of EBV DNA and expression patterns of EBV-related genes in human tonsil tissue explants infected with EGFP-EBV. Cell-free supernatants and cells collected from culture medium were used for quantification of EBV DNA by real-time PCR assay and quantification of viral mRNA by real-time RT-PCR assay. Data are presented as mean ± standard error of the mean. **A**) Plots of accumulated EBV DNA in cell-free culture medium after 12 days post-infection (*n* = 8). **B**) Plots of accumulated EBV DNA in cells from medium (*n* = 2). **C**) Levels of EBV-related genes expressions in cells at 3 and 24 days post-infection (*n* = 3).

We tested the ability of EGFP-EBV to replicate in human tonsil tissues in the same way as B95–8. Levels of EBV DNA in culture supernatants of EGFP-EBV-infected tissues as primary data were measured for each time point. EBV DNA levels gradually decreased and were minimal on days 12–15 post-infection, then steadily increased (data not shown). Productive infection was documented by the kinetics of EBV DNA accumulation in culture supernatants of infected tissues using real-time quantitative PCR (**Fig. 2A**), with peak levels between 2.1×10^4^ and 2.5×10^6^ genome-equivalents/ml of culture supernatant (median, 3.5×10^5^ genome-equivalents/ml of culture supernatant) at day 24 post-infection in tissues infected with EGFP-EBV (*n* = 8). Increasing levels of EBV over time were also detected by measuring cell-associated DNA (**Fig. 2B**), with peak levels between 1.4×10^5^ and 4.1×10^5^ genome-equivalents/µg DNA at day 24 post-infection (*n* = 2).

Expressions of viral genes in cells collected from culture medium of EGFP-EBV infected tissues are shown in **Figure 2C**. Expressions of lytic cycle genes and increased levels of genes expression were subsequently observed at day 24 post-infection (*n* = 3). Use of recombinant EGFP-EBV did not influence EBV gene expression compared to that in B95–8.

### Histological analyses in EBV-infected human tonsil tissues

**Figure 3:**
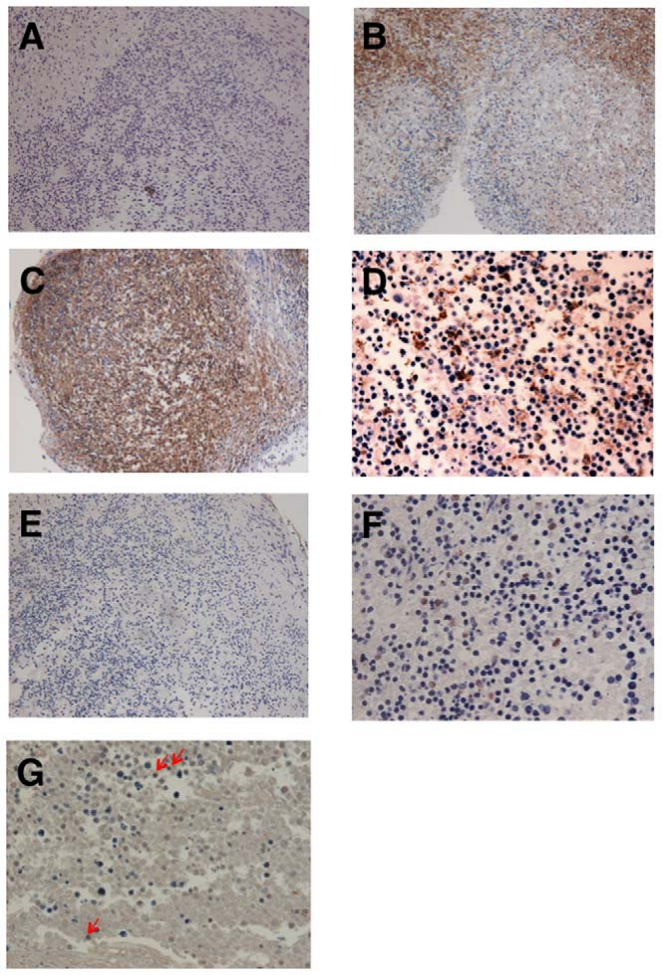
Histological analyses of tonsil tissues infected with B95–8 using immunostaining and *in situ* hybridization with EBER-RNA. Samples were obtained from EBV-infected tissues at 24 days post-infection. Pictures are representative of results from four experiments. **A**) Low-power view of tonsillar lymphoid tissue (HE stain). Magnification, ×100. **B**) Low-power view of CD3^+^ lymphocytes in an interfollicular area (CD3 stain). Magnification, ×100. **C**) Low-power view of CD20^+^ lymphocytes in a follicular area (CD20 stain). Magnification, ×100. **D**) High-power view of anti-follicular dendritic cell (FDC)^+^ in a follicular area (anti-FDC stain). Magnification, ×400. **E**) Low-power view of EBER^+^ lymphocytes (EBER ISH stain). Magnification, ×100. **F**) High-power view of EBER^+^ lymphocytes in an interfollicular area (EBER ISH stain). Magnification, ×400. **G**) High-power view of BZLF1^+^ lymphocytes (arrows) in an interfollicular area (BZLF1 stain). Magnification, ×400.

Histological analysis showed that tissues retained their gross morphology, even at day 24 post-infection (**Fig. 3A**, *n* = 4). Immunohistochemical analysis performed by staining paraffin-embedded sections of tissue blocks with anti-CD3, anti-CD20, and anti-FDC mAbs revealed the presence of T cells (**Fig. 3B**), B cells (**Fig. 3C**), and FDCs (**Fig. 3D**). B cells were concentrated in follicular areas (**Fig. 3C**), while T cells (**Fig. 3B**) were confined to interfollicular areas. FDCs were detected in follicular areas (**Fig. 3D**). EBER-positive cells were detected, mainly in the interfollicular areas (**Fig. 3E**). EBER-positive cells represented a mixture of cells ranging from large lymphoid blasts to small lymphoid cells (**Fig. 3F**). No morphological evidence suggested EBV infection of cells other than lymphoid cells. BZLF1, a hallmark of lytic infection, was also detected in lymphoid cells (**Fig. 3G**), while EBNA2 and LMP1 were not detected in tissue samples.

### Target cells of primary EBV infection in human tonsil tissues

**Figure 4:**
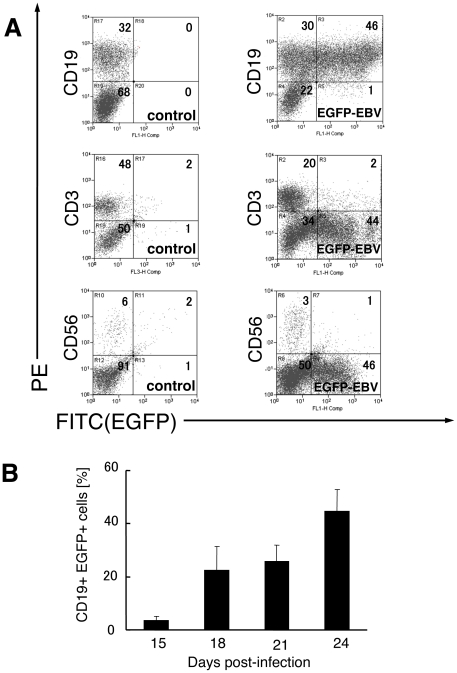
Phenotype of EGFP^+^ cells in human tonsil tissue infected with EGFP-EBV. At 15–24 days post-infection, single cell suspensions were dissected from tissue blocks. Cells were stained with phycoerythrin (PE)-labeled anti-CD19, CD3, CD56 monoclonal antibodies, then analyzed by flow cytometry. **A**) A representative experiment is shown (*n* = 4). Density plots represent CD19, CD3, CD56 versus EGFP for control and EGFP-EBV-infected tissues at 24 days post-infection. Numbers in quadrants indicate percentages of lymphocytes for each surface immunophenotype. **B**) Mean proportion of EGFP^+^ CD19^+^ cells among CD19^+^ cells at 15–24 days post-infection. Average data were obtained from 27 blocks of tissue derived from four donors. Results represent mean ± standard error of the mean. ctrl, control (uninfected tonsil tissues).

**Figure 5:**
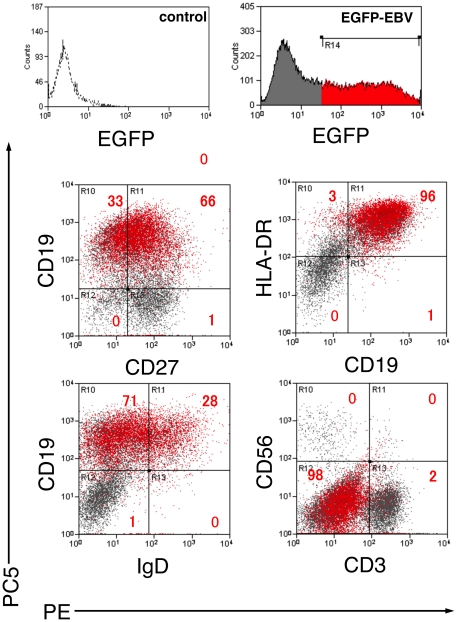
Phenotype of EGFP^+^ cells in human tonsil tissue infected with EGFP-EBV using flow cytometry. Samples were obtained from EBV-infected tissues at 24 days post-infection. EGFP^+^ (red) and EGFP^−^ (grey) MNCs were gated and plotted on quadrants as PE-labeled and PC5-labeled surface antigens. A representative experiment is shown (*n* = 2). Numbers in quadrants indicate percentages of EGFP^+^ lymphocytes for each surface immunophenotype.

To quantify and identify EBV-infected cells in tonsil tissues, we investigated the lineage of EGFP^+^ cells prepared from tissue blocks infected with EGFP-EBV by flow cytometry using mAbs directed against several lymphocyte membrane antigens. GFP signals were not clearly detected before day 15, and the initial EBV-infected cells could not be determined (data not shown). Collected cells were stained with 7-AAD at day 24 in three experiments and 7-AAD-positive cells comprised 2.3±1.6% of the lymphocyte population gated by standard forward and side scatter profiles (data not shown). **Figure 4A** shows a representative experiment with results (*n* = 4) indicating that the majority of EGFP^+^ cells were CD19^+^ cells, with a very limited proportion of other types of lymphocytes. **Figure 4B** represents the kinetics in the proportion of EGFP^+^ CD19^+^ cells among all CD19^+^ cells at 15–24 days post-infection (*n* = 4). Mean proportions of EGFP^+^ CD19^+^ cells increased over time, with frequencies of 3.7±1.3%, 22.5±9.1%, 25.7±6.2%, and 40.8±11.3% at days 15, 18, 21, and 24 day post-infection, respectively. These data indicate that EBV mainly infected B cells in tonsillar tissue explants. Furthermore, EGFP^+^ cells were closely examined to determine the phenotype of EBV-infected cells. A representative result is shown in **Figure 5** (*n* = 2). Most EGFP^+^ cells were CD3^−^ CD56^−^ CD19^+^ HLA-DR^+^, and both naïve (CD19^+^IgD^+^, mean 22.6±6.0% of EGFP^+^ cells) and memory (CD19^+^ CD27^+^, mean 73.5±7.5% of EGFP^+^ cells) B cells were detected.

### Inhibition of EBV replication by ACV in human tonsil tissues

**Figure 6:**
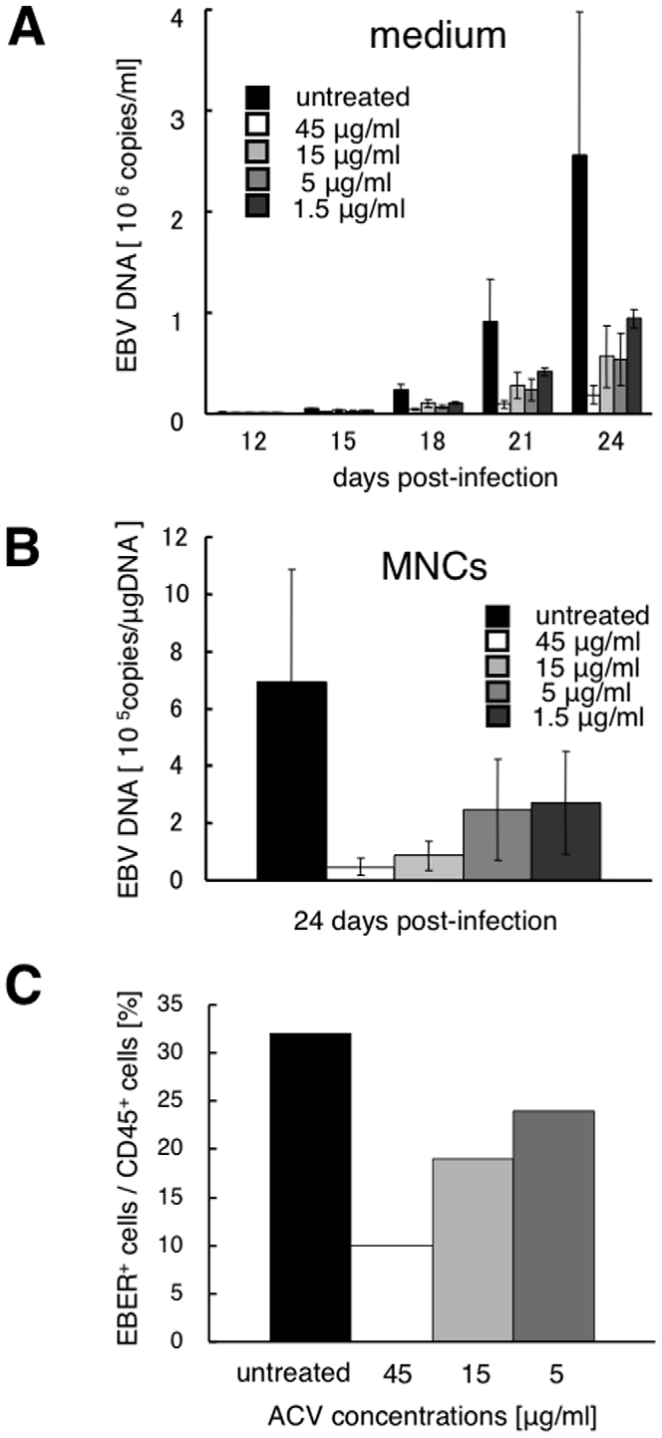
Inhibition by acyclovir (ACV) of EBV replication in human tonsillar tissues explants. Human tonsillar tissues were infected with B95–8 and treated with ACV at various concentrations. Antiviral activity of ACV was evaluated by comparing viral replication in ACV-treated tissues with that in untreated donor-matched control tissues. Data are presented as means ± standard errors of mean. **A**) Kinetics of EBV replication were measured by real-time PCR assay for viral DNA in culture medium (*n* = 3). **B**) EBV DNA was quantified in cells at 24 days post-infection (*n* = 3). **C**) Comparison of the number of EBV-infected cells in the presence or absence of ACV at 24 days post-infection using FISH assay (*n* = 1).

Finally, we investigated the anti-EBV activity of ACV in infected tonsil tissues. Blocks of tonsil tissues were cultured with medium containing various concentrations of ACV. The amount of EBV DNA was measured in cell-free culture medium and in cells collected from culture medium. EBV replication was suppressed by ACV treatment in a dose-dependent manner (**Fig. 6A**). Mean accumulations of EBV DNA in culture medium at day 24 post-infection were 1.9±0.9×10^5^, 5.7±3.1×10^5^, 5.4±2.6×10^5^, 9.9±5.3×10^5^, and 2.6±1.4×10^6^ genome-equivalents/ml in tissues treated with 45, 15, 5, 1.5, and 0 µg/ml of ACV, respectively (*n* = 3). Reduction rates in total production of EBV DNA accumulated in culture medium at day 24 post-infection were 90±4%, 76±7%, 77±3%, and 64±2% in tissues treated with 45, 15, 5, and 1.5 µg/ml of ACV (*P*<0.01), respectively. Suppression of EBV replication was also documented by measurement of EBV DNA in cells from culture medium (**Fig. 6B**). Mean levels of EBV DNA in these MNCs were 4.7±3.1×10^4^, 8.7±5.2×10^4^, 2.5±1.8×10^4^, 2.7±1.8×10^5^, and 6.9±3.9×10^5^ genome-equivalents/µg DNA at day 24 post-infection in tissues treated with 45, 15, 5, 1.5, and 0 µg/ml of ACV, respectively (n = 3). Moreover, the number of EBV-infected cells was compared in the presence or absence of ACV using FISH assay. The percentage of EBER^+^ cells among CD45^+^ cells was decreased at day 24 post-infection in a dose-dependent manner (10% with 45 µg/ml of ACV, 19% with 15 µg/ml, 24% with 5 µg/ml, and 32% without ACV, *n* = 1) (Fig. 6C).

## Discussion

Viral infection models using human lymphoid tissues have the advantages of enabling culturing while maintaining tissue cytoarchitecture (including major lymphocyte subtypes and the follicular-dendritic cell network) and supporting productive virus infection without exogenous activation and stimulation. These models have been reported as useful for analyzing the pathogenicity of viruses that mainly infect lymphoid tissues [16]. In a study of the herpesvirus family, Grivel *et al.* used a tonsillar infection model to investigate cellular tropism and the pathogenic effects of HHV-6, demonstrating that HHV-6 infects efficiently without exogenous stimulation and that T-lymphocytes are the main cells infected [19]. The present study established an experimental EBV infection model with a culture system using tonsil tissues and confirmed that the amount of EBV-DNA in medium and in cells increased over time. The increase in DNA in this infection model was pronounced from ≥12 days post-infection, with the amount of virus starting to increase later using EBV than in tonsillar infection models using HHV-6 or HHV-7 [18], [19]. This was not regarded as a problem with the tonsillar infection model, but rather as resulting from differences in viral proliferation; however, establishment of an EBV infection model may be more difficult for this reason. Yajima *et al.* infected humanized NOG mice intravenously with EBV and reported that EBV-DNA in blood increased after 3–4 weeks [15]. Changes in the amount of DNA in our infection model were not inconsistent with those results. We also investigated expression of EBV-related genes in cells obtained from our infection model, revealing expression of lytic genes and increased levels of gene transcripts over time, in addition to latent genes expressed as latency type III. These data show that EBV produces both latent and productive infections in a tonsillar infection model. Several histological studies of IM tonsils have shown the presence of B cells expressing latency types III, II, and I, as well as lytic genes [32], [33]. In the present EBV-infection model, EBV *de novo* synthesis was not shown directly. However, several results suggested that at least some of the infected cells in this model gave rise to progeny virus: (1) the amount of EBV DNA in both culture media and cells markedly increased after day 12 post-infection, (2) immediate early gene BZLF1 was detected in lymphoid cells both in the immunohistochemical analysis and in the quantification of mRNA expression, and (3) ACV treatment reduced the levels of EBV DNA. This model may represent early events in primary EBV infection, before the emergence of IM symptoms, rather than a stable latent infection. Taken together, the tonsillar infection model is useful for analyzing the pathology of primary infection with EBV.

EBV is believed to be transmitted orally at oropharyngeal sites [34]. For this reason, numerous histological studies of IM tonsils have examined viral-cell interactions during primary infection [32], [35], [36]. These investigations have been unable to detect EBV infection of the tonsillar epithelium, with most reports stating that the main infected cells are B cells [32], [35], [36], [37]. Our histological analysis also suggested B-cell infection. Flow cytometric analysis also showed that B cells were mainly infected, supporting the findings of previous studies. The events involved in virus colonization of the B-cell system during primary infection remain poorly understood. Various studies have looked at tonsillar EBV-infected B-cell subsets in healthy virus carriers [38], [39], [40]. Joseph *et al.* used IgD mAb from the tonsils of healthy virus carriers to sort each B-cell subset and performed limiting-dilution DNA PCR on each subset, detecting EBV in both naïve (IgD^+^) and memory B cells [38].

Meanwhile, the only report on EBV-infected B-cell subsets in tonsils obtained from patients with IM has been the recent study by Chaganti *et al.* [36]. They sorted each B-cell subset using CD27, IgD, and CD38 mAbs from tonsils they obtained and performed quantitative PCR on each subset, reporting that the amount of DNA was greatest in CD38^+^ cells, followed by memory cells (CD27^+^) and finally naïve (IgD^+^ CD27^−^) B cells. We investigated the lineage of EGFP^+^ cells dissected from tissue blocks infected with EGFP-EBV by flow cytometry using mAbs directed against several lymphocyte membrane antigens and the proportion of EBV-infected B cells was higher among memory cells (CD27^+^) than among naïve cells (IgD^+^), consistent with findings from the above study. This showed that the tonsillar infection model is useful for analyzing EBV-infected lymphocyte subsets. Further observation of changes in subsets over time as well as of additional surface or intracellular molecules, such as cell adhesion markers and chemokine receptors, will enable us to characterize and examine the function of EBV-infected cells.

Conversely, tissue culture models show a number of limitations. The first is that wide differences in proliferative capacity within tissue exist between donors. In practice, even when experiments have been performed under the same protocol, differences of around 100-fold have occurred in amounts of DNA between tissues. The same phenomenon has also been observed in tonsillar infection models using other viruses [19], [41]. This is probably because the structure and cell composition of tonsil tissue vary greatly among donors. The second limitation that can be raised is that tissues deteriorate as a result of long-term culture. We cultured cells for 4 weeks, but regarded culture for longer than this period as difficult due to deteriorations observed in tissue samples. Histological investigation of tissue segments also showed that although the structures were maintained, the number of lymphocytes decreased significantly. The third limitation is that investigation of EBV-specific immune responses was difficult in this model. Symptoms of IM are believed to be closely related to the specific immune responses of the host to EBV infection [1]. With respect to specific immune response in tonsillar infection models, antibody response to antigen load with antigens such as diphtheria and tetanus toxoids has been reported [42]. However, IM symptoms appear as late as 4–6 weeks after viral transmission *in vivo* [2]. Since tissue deterioration makes culture for longer periods than this difficult, evaluation of specific immune responses was considered difficult.

Treatment of IM and PTLD with regular antiviral agents is not recommended [1], but antiviral agents may be required in cases such as immunocompromised patients with severe IM or HIV-infected patients with oral hairy leukoplakia, and novel antiviral agents need to be developed for treatment in such cases. ACV is known to be a guanosine nucleoside analog with activity against several types of α-herpesvirus. ACV inhibits virus-associated DNA polymerase, suppressing virus replication. Although some reports have indicated that ACV is effective against EBV *in vitro* [43], [44], clear efficacy in actual clinical practice has not been established [1], [2]. Using the tonsillar infection model, we showed that ACV exerts a dose-dependent antiviral action. For previous evaluations of *in vitro* efficacy, EBV-infected cell suspension cultures have been used [43], [44]. Actual EBV infection, however, occurs in the oropharyngeal lymphoid tissues [2], [34], so use of a tonsillar infection model may more closely reproduce the *in vivo* environment. The tonsillar infection model was also regarded as applicable to the screening of novel antiviral agents.

In this study, we established an EBV infection model using human tonsil tissues. EBV infection and proliferation in this model could be observed without the need for any special exogenous stimulation. Use of flow cytometry also enabled qualitative and quantitative analysis of infected cells. This model has potential for use in the pathological analysis of local tissues at the time of primary infection, as well as for screening novel antiviral agents.
